# Indian Academy of Neurology: The Dasgupta Oration 2009, Neural regeneration with stem cells: Rebooting the brain

**DOI:** 10.4103/0972-2327.56311

**Published:** 2009

**Authors:** Alok Srivastava

**Affiliations:** Department of Hematology, Christian Medical College, Vellore, India. E-mail: aloks@cmcvellore.ac.in

It has long been recognized that injured neurons in the central nervous system (CNS) have a limited capacity to regenerate. Neural injury from trauma, ischemia, or degeneration is therefore devastating for such patients and their families. The success of conventional drug therapy has also been limited. Given this background, it is understandable why there is so much enthusiasm and excitement about the use of stem cells for neural regeneration. It is now clear that stem cells in the adult are not confined to only a few organs and neither are they necessarily restricted in their ability to differentiate into cells other than those of the organs they come from. In other words, they may still retain some of their plasticity.[1]

## Stem Cell Therapies for Neurological Disorders

For some years now therefore attempts have been made to use the more easily accessible adult stem cells for treating various neurological diseases. These include models of spinal cord injury, traumatic brain injury, brain ischemia, and degenerative brain disorders. Several clinical trials have been undertaken. The cells that have been most commonly employed are the bone marrow–derived hematopoietic stem cells, mostly autologous. There are reports of other relatively easily accessible progenitor cells, such as cord blood stem cells and fetal neural stem cells, also having been used in some studies. A few researchers have used mesenchymal stem cells, in the allogeneic setting, as well. In both animal models and some early human studies, there have been encouraging responses, suggesting that enhanced functional recovery may be possible.

While there is much promise, there is no evidence as yet that any of these treatments are clearly beneficial and so further studies are needed. The path to clinical use of stem cells has been defined in the Guidelines for Clinical Translation of Stem Cell Research published recently (http://www.isscr.org/clinical_trans/pdfs/ISSCRGLClinicalTrans.pdf) by the International Society for Stem Cell Research.[2] There are four critical parts to this – choosing the most suitable well-defined cells prepared under cGMP (current good manufacturing practice) conditions, preclinical evaluation in appropriate animal models, clinical assessment in carefully designed and controlled studies, and ensuring that all this is done while paying full attention to ethical issues and social justice.

There are special challenges in applying these guidelines to neurological disorders because not only is there a lack of good models but the assessment of response, particularly behavioral, in animal models can be difficult. If results in small animal studies look promising, then one needs to consider further evaluation in larger animals before going on to human studies. Many studies using adult autologous bone marrow–derived cells in neurological disorders have been reported, almost all of them with small numbers of patients.[3–5] The first study using embryonic stem cell–derived tissue was sanctioned by the US FDA in early 2009[6] for patients with spinal cord injury. Prior to this, in late 2008, a study on the use of fetal brain–derived neural stem cells in amyotrophic lateral sclerosis had received sanction. It is important to note that both these studies are currently on hold by the FDA (August, 2009) for review of safety issues.

From the safety perspective, the follow-up period needs to be prolonged in all these studies so that late complications, if any, can be noted. It is important to recognize that unlike other drug therapies, the product used in this therapy – stem cells – do not have a defined half-life. This fact has been remarkably driven home by the report of multifocal neural tumors arising from donor–derived cells in an Israeli child with ataxia telangiectasia who had been treated with fetal neural stem cells in Russia 4 years earlier.[7] Apart from using unmodified stem cells for organ regeneration, given the tropism of neural stem cells for malignant gliomas, such cells can also be used to target cytolytic therapy such as intra-tumor herpes simplex virus thymidine kinase.[8]

## Possibilities in India - Challenges to Clinical Translation of Stem Cell Research

There are many factors that favor the scope for good clinical research with stem cells in India. These include the facts that there are a large numbers of patients who can benefit from this form of treatment, as many do not have access to the kind of supportive care that is needed to for managing long-standing disabilities. Innovative therapies are therefore a greater clinical need here. We also have large numbers of well-trained clinicians familiar with the conduct of clinical trials. The basic technology needed for preparing commonly used adult autologous stem cells for such studies are also well established in many centers in this country.[9] Yet the actual number of good clinical trials for neurological disorders is small. The reasons for this may be that not enough clinicians are convinced that this form of therapy is worth pursuing; alternatively, interested researchers may lack the technological ability to prepare the required cells for this therapy or just may not have the appropriate infrastructure for conducting clinical trails.

## Clinical Translation in India: Guidelines for Stem Cell Research and Regulating Therapy in India

While there are few well-designed clinical trials with stem cells in India for neurological disorders, many patients are unfortunately getting treated with stem cell therapies outside of clinical trials, often at very significant costs. In fact, this raises another very critical issue in this field. While on one side, the majority of neurologists perhaps think that there is not enough data to justify major human studies, there are others who are offering some of these therapies outside of clinical trials. The question therefore arises regarding the kind of clinical response data that should be available before a particular therapy can be considered to be the standard of care. This answer is best provided by a body of specialists in the field.

There is also the issue of regulation of stem cell trials in India. In general, in India, all clinical trials are regulated by the Drugs Controller General of India (DCGI). However, we must appreciate that stem cell trials require special understanding of the issues involved; these are very different from that in trials with small molecules and even the DCGI will need to acquire suitable expertise. While the guiding philosophy should be to promote trials of therapies with stem cells, it must be done taking into account the scientific and ethical aspects relevant in the social context of this country. The Indian council of Medical Research (ICMR) and the Department of Biotechnology (DBT) of the Ministry of Science and Technology, Government of India, have put together the guidelines for stem cell research in India, which provides the principles that need to be followed (http://icmr.nic.in/stem_cell/stem_cell_guidelines.pdf). The challenge now is to ensure that this is actually implemented.

In conclusion, it is possible that stem cell-based therapies could provide completely new possibilities for the treatment of many, so far, untreatable neurological disorders. However, for that to happen, forward planning and coordination is needed between the various stakeholders – interested physicians, funding agencies, regulators, and industry – so that desperate patients are provided opportunities for innovative therapies in a scientific and ethical way, without being exploited.
